# Supplementary material for: Molecular architecture and electron transfer pathway of the Stn family transhydrogenase
Source: Nat Commun. 2023 Sep 7;14:5484. doi: 10.1038/s41467-023-41212-x (PMC10482914; doi:10.1038/s41467-023-41212-x)
Supplement: Supplementary file 1 — Supplementary Information [file 41467_2023_41212_MOESM1_ESM.pdf]

Supplementary Information

Molecular architecture and electron transfer pathway of the Stn family transhydrogenase

Authors:

Anuj Kumar[1,2†], Florian Kremp[2†], Jennifer Roth[2], Sven A. Freibert[1,3,4], Volker Müller[2*], Jan M. Schuller[1*]

Affiliations:

[1]SYNMIKRO Research Center and Department of Chemistry, Philipps-University of Marburg, Marburg, Germany.

[2]Department of Molecular Microbiology & Bioenergetics, Institute of Molecular Biosciences, Johann Wolfgang Goethe University, Frankfurt am Main, Germany.

[3]Institut für Zytobiologie im Zentrum SYNMIKRO, Philipps-University of Marburg, Marburg, Germany.

[4] Core Facility "Protein Biochemistry and Spectroscopy", Marburg 35032, Germany

† Equal contribution

*Correspondence to: vmueller@bio.uni-frankfurt.de and jan.schuller@synmikro.uni-marburg.de

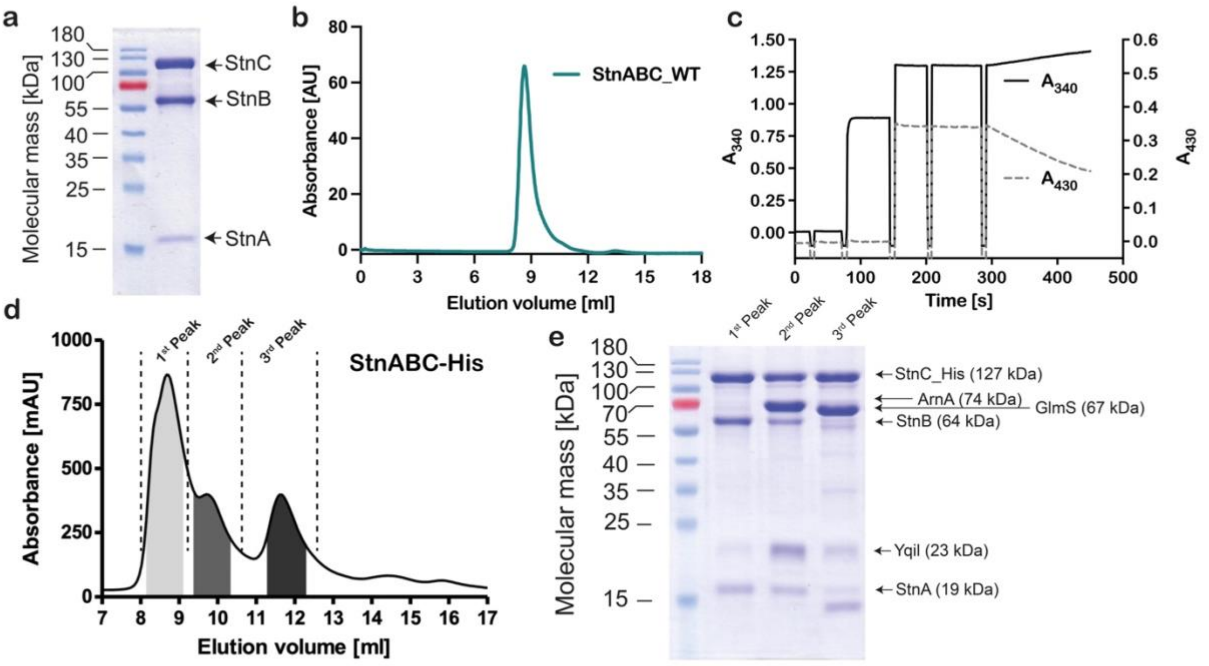

Supplementary Fig. 1 | Properties of native StnABC and heterologously produced StnABC-His. (a) 10 µg of purified StnABC complex from *S. ovata* was separated using SDS-PAGE. (b) Elution profile of purified StnABC complex from *S. ovata* during size exclusion chromatography on “Superdex 200 Increase™ 10/300”. (c) Measurement of simultaneous NADPH-dependent Fd_{ox} and NAD^+ reduction catalysed by the StnABC from *S. ovata*. (d) Separation of StnABC-His from contaminating protein by size exclusion chromatography on “Superdex 200 Increase™ 10/300”. (e) 10 µg of protein samples of peaks 1, 2 and 3 from (d) were separated by SDS-PAGE.

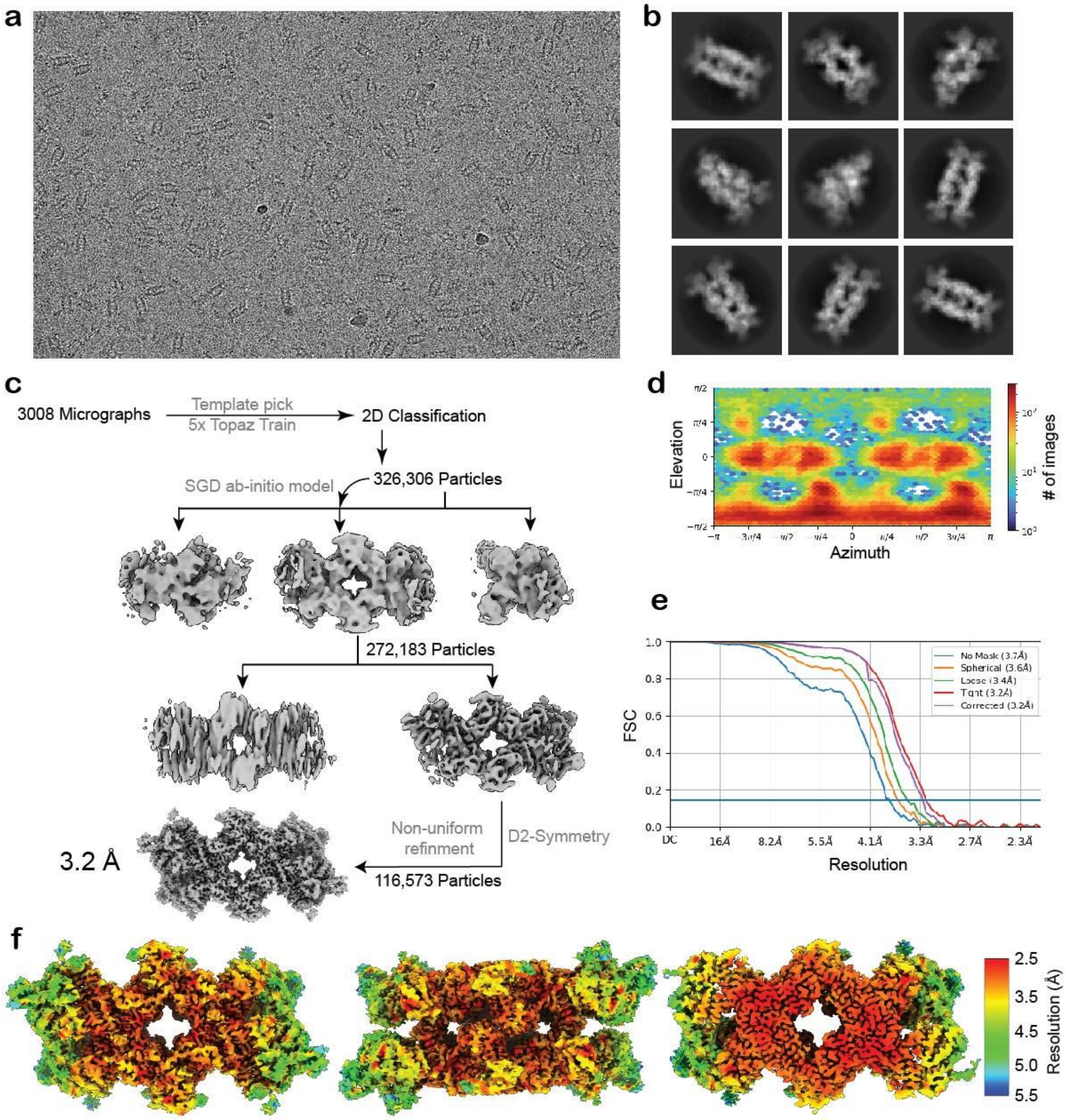

Supplementary Fig. 2 | Cryo-EM data collection and analysis of StnABC complex in the isolated or purified state of the enzyme (StnABC$_{S1}$ state). (a) A representative cryo-EM micrograph showing StnABC tetrameric particles. (b) Reference-free 2D class averages revealing different views of the complex. (c) Overview of the cryo-EM data-processing scheme. (d) Angular distribution of the particles used for the final round of refinement. (e) Plot showing the global resolution. (f) Local resolution as calculated by CryoSparc mapped on the refined density (left, middle: front and side view, right: cut-open view of central section).

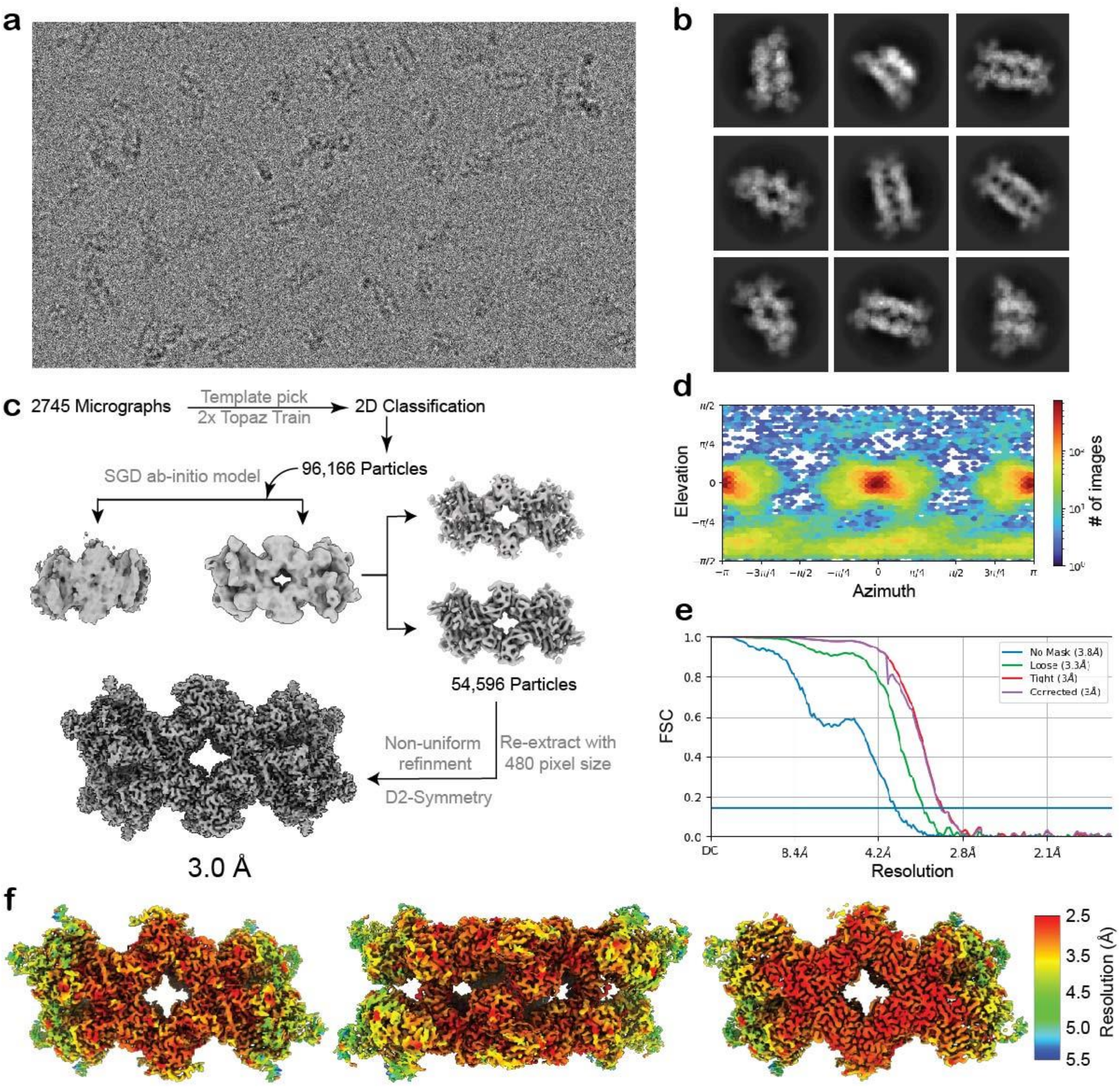

Supplementary Fig. 3 | Cryo-EM data collection and analysis of StnABC complex in the presence of added cofactors (FAD, FMN) and substrates (NADPH, NAD^+, Fd) ($StnABC_{S2}$ state). (a) A representative cryo-EM micrograph showing single StnABC particles. (b) Reference-free 2D class averages revealing different orientations of the complex. (c) Overview of the cryo-EM data-processing scheme. (d) Angular distribution of the particles used for the final round of refinement. (e) Plot showing the global resolution. (f) Local resolution as calculated by CryoSparc mapped on the refined density (left, middle: front and side view, right: cut-open view of central section).

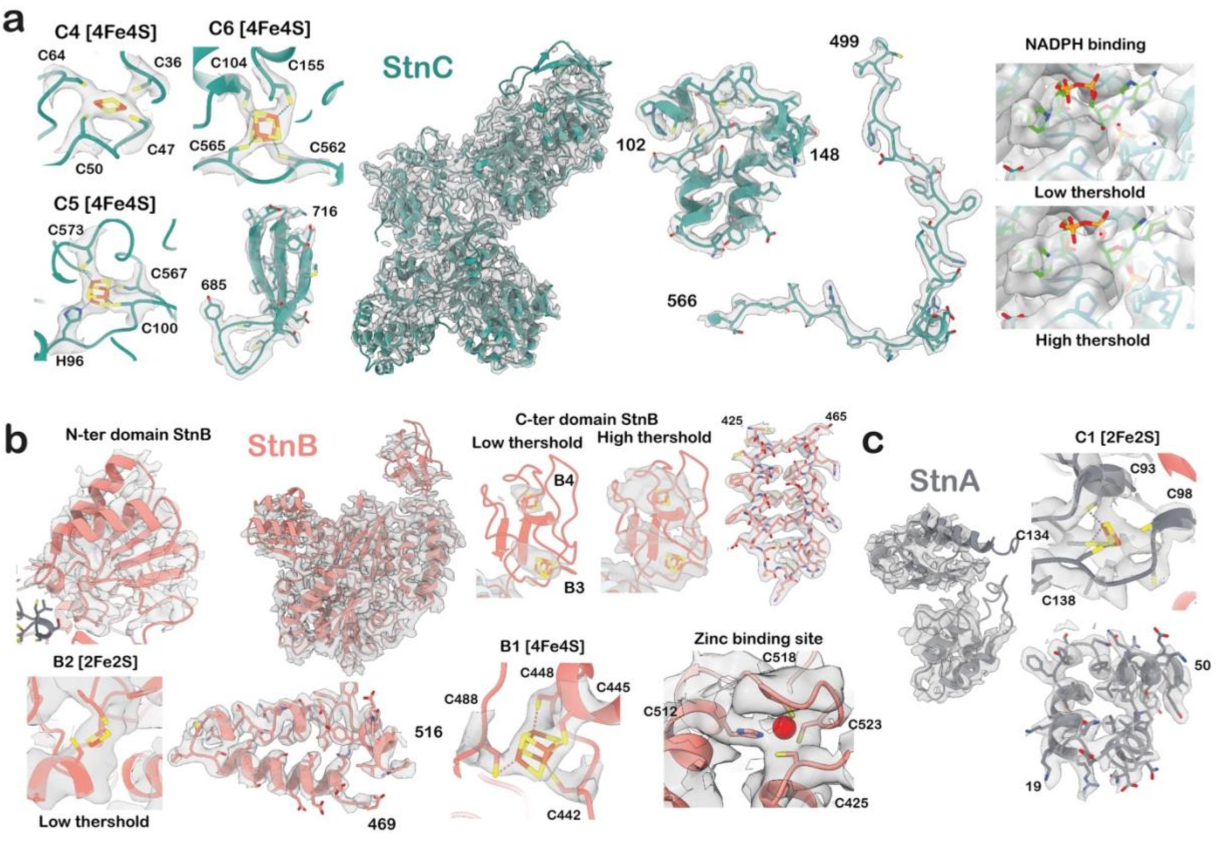

Supplementary Fig. 4 | Cryo-EM density and model quality. Representative regions of the StnC, StnB, and StnA subunit and their surrounding electron density maps are shown. **(a)** The StnC subunit formed the core of the complex and relatively had better resolution compared to the peripheral StnAB subcomplex. The NADPH binding was found to be transient due to the weak and diffusive density observed in the cryo-EM structure of the $StnABC_{S2}$ state. As a result, the NADPH modelling was performed based on the crystal structure of the NfnAB complex with bound NADPH. Local density around NADPH is shown at low and high thresholds. **(b)** For StnB N-ter and C-ter, precise atomic modelling was not possible as these regions exhibited extensive flexibility. The electron density for the B2 [2Fe2S]-cluster in the N-ter and B3-B4 [4Fe4S]-clusters at the C-ter of the StnB subunit is shown at low and high thresholds. **(c)** Regions of StnA subunit encased around cryo-EM density map. All maps are displayed as a surface using a contour level of up to 2 Å around the atoms. The colour code used corresponds to Fig. 1.

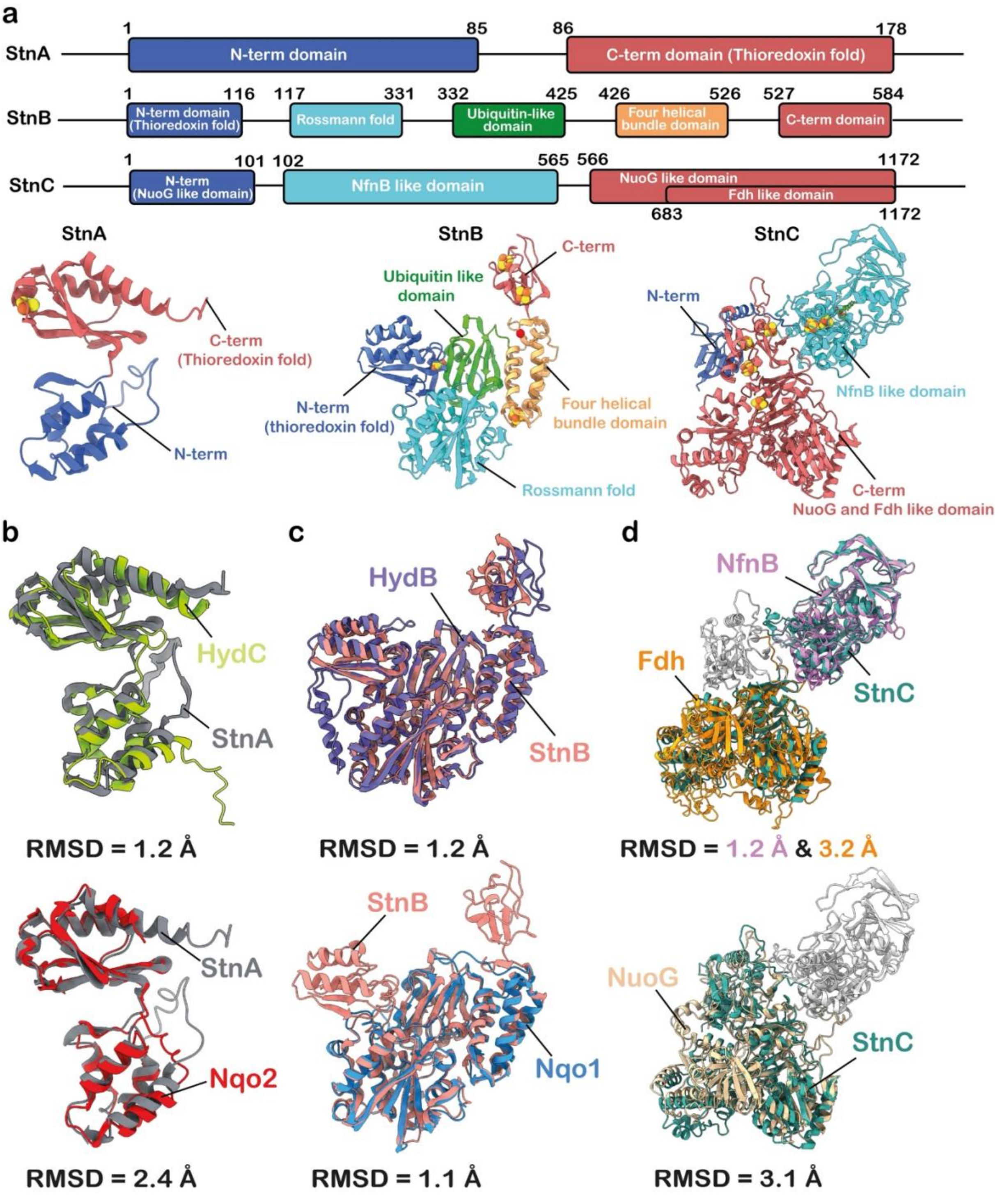

Supplementary Fig. 5 | Domain dissection of StnABC complex and structural alignment with its closest homologs. (a) Domain distribution is coloured according to different folds in the StnABC complex. The StnA subunit consists of an N-terminal domain with a flexible C-terminal thioredoxin-like fold consisting of a binuclear [2Fe2S]-cluster. The StnB, being very similar to HydB, also has an N-terminal thioredoxin-like fold containing a [2Fe2S]-cluster. This is followed by a Rossmann fold harbouring the FMN, a ubiquitin domain, a four helical

bundle domain with one [4Fe4S]-cluster, and the extension from the helical bundle is the highly flexible C-terminal bacterial Fd-like domain consisting of two [4Fe4S]-clusters. The StnC is highly modular in nature, the small N-terminal portion (1-101) combines with the entire C-terminal part (566-1172) to form a NuoG-like domain containing four [4Fe4S]-clusters and one [2Fe2S]-cluster. The middle domain (102-565) is homologous to the NfnB subunit, harbouring the non-bifurcating FAD and two [4Fe4S]-clusters. The C-terminal alone cognates with formate dehydrogenase. (b) The StnA is highly similar to HydC from *T. kivui* (PDB ID - 8A6T) or NADH:quinone oxidoreductase subunit 2 (Nqo2) from *T. thermophilus* (PDB ID - 6Q8W) with an RMSD value of 1.2 Å and 2.4 Å, respectively. StnB subunit structurally aligns well with HydB from *T. kivui* (PDB ID - 8A6T) and Nqo1 from *T. thermophilus* (PDB ID - 6Q8W) with an RMSD of 1.2 Å and 1.1 Å, respectively. Whereas the StnC subunit has three domains which are similar to NfnB from *P. furiosus* (PDB ID – 5JCA) (RMSD – 1.2 Å), FdhF from *D. gigas* (PDB ID – 1H0H) (RMSD – 3.2 Å), and NuoG from *E.coli* (PDB ID – 7NZ1) (RMSD – 3.1 Å).

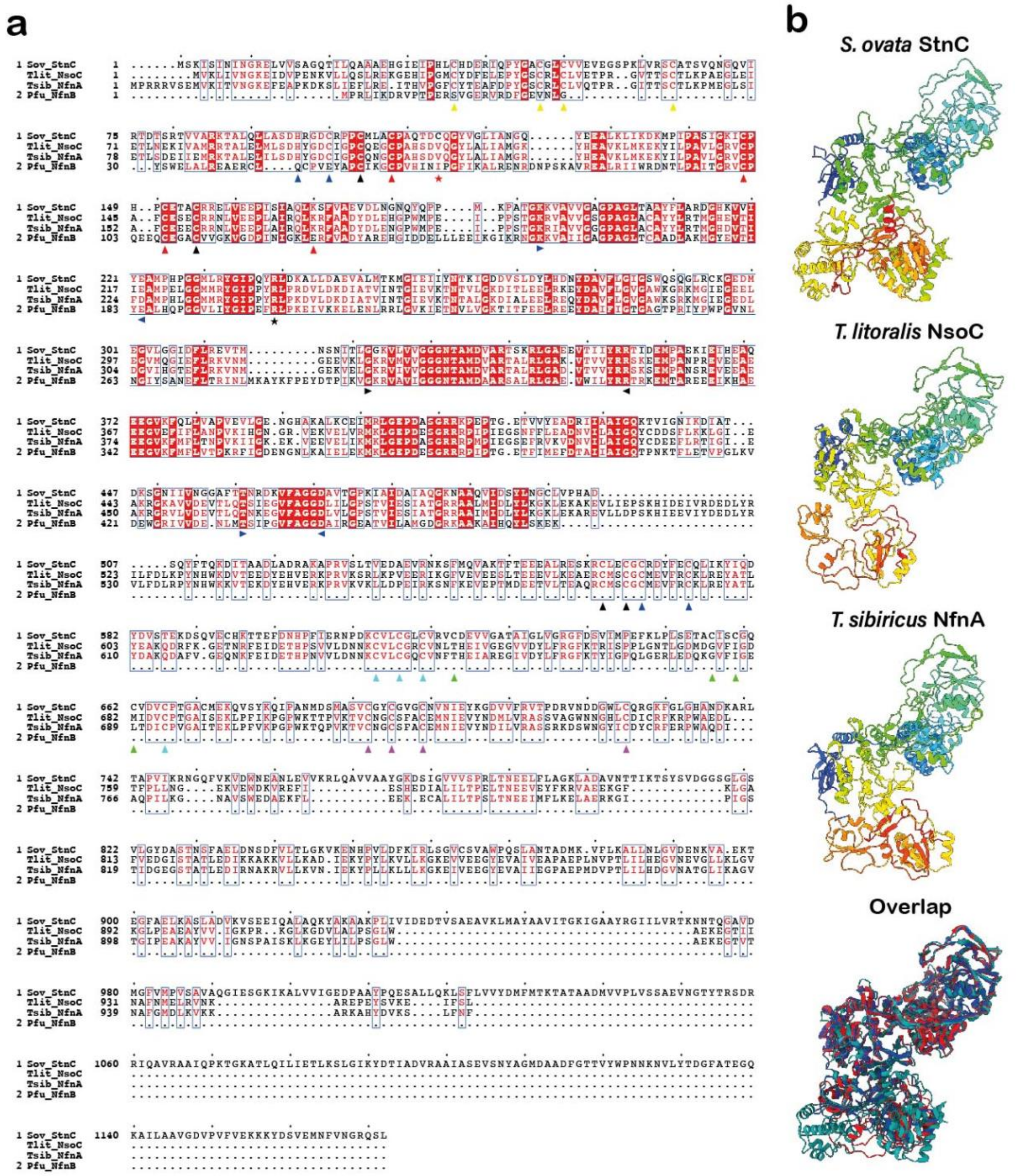

Supplementary Fig. 6 | (a) Alignment of the large subunit of electron bifurcating transhydrogenases. (a) StnC of *S. ovata* was aligned with its homologs of *T. litoralis* (NsoC), *T. sibiricus* (NfnA) and NfnB of *P. furiosus*. Red triangles indicate [4Fe4S]-cluster C7, black triangles indicate residues responsible for the binding of [4Fe4S]-cluster C6, blue triangles indicate residues responsible for the binding of [4Fe4S]-cluster C5, yellow triangles point to the residues coordinating the [2Fe2S]-cluster C4, cyan triangles indicate residues responsible for the binding of [4Fe4S]-cluster C3, green triangles indicate residues responsible for the

binding of [4Fe4S]-cluster C2 and magenta triangles indicate residues responsible for the binding of [4Fe4S]-cluster C1. Blue sideways triangles indicate the FAD binding site, whereas black sideway triangles indicate the NADPH binding site. A black asterisk indicates the strictly conserved residue R239. The Red asterisk indicates residue C114, which is not conserved among the homologs. (b) Alphafold models of *T. litoautotrophicus* (NsoC), *T. sibiricus* (NfnA) and its superposition with StnC subunit. The C-terminal formate dehydrogenase domain from NsoC and NfnA seems to be partial, whereas StnC contains a fully intact FdhF-like domain.

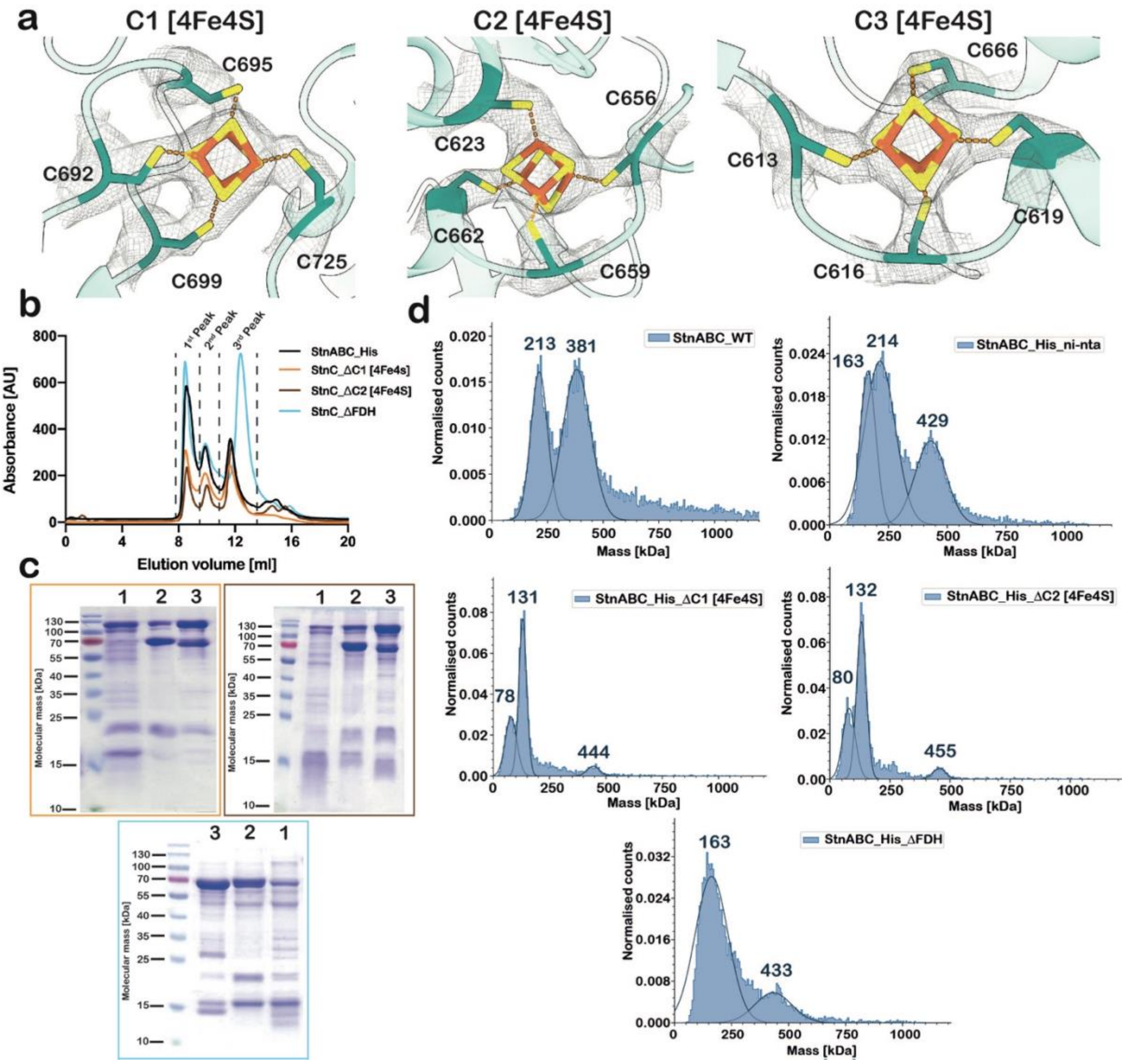

Supplementary Fig. 7 | Mutational analysis of C1 and C2 clusters, and the Fdh domain in StnC. (a) C1, C2, and C3 [4Fe4S]-clusters with their respective ligand sphere wrapped around their respective cryo-EM density (in mesh). (b) Size exclusion curves of the purified StnABC with ΔC1, ΔC2, and ΔFdh domain variants. Mutating the C1/C2/Fdh domain had a destabilizing effect, causing the protein to be purified as a partial complex. (c) 10 µg of protein samples of peaks 1, 2 and 3 from (b) were separated by SDS-PAGE. (d) Mass photometry measurements confirm the presence of partial complex for the variants ΔC1, ΔC2, and ΔFdh domain when compared to the WT StnABC complex, which existed in the dimeric and monomeric population.

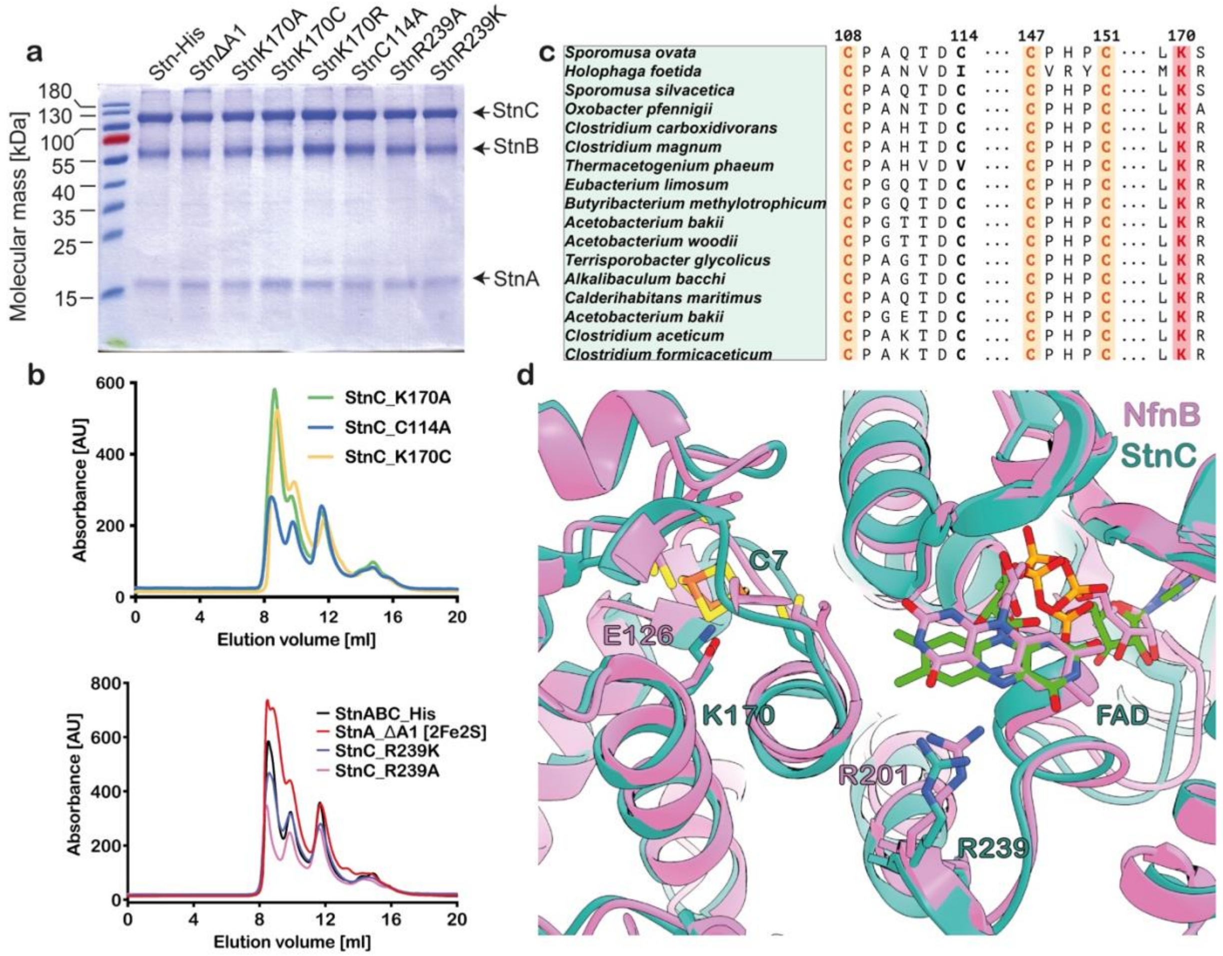

Supplementary Fig. 8 | Purification and characterization of mutants modulating the activity of the StnABC complex. (a) Purified StnABC variants separated using SDS-PAGE. (b) Elution profile of overproduced and IMAC-enriched StnABC-His mutants in size exclusion chromatography on “Superdex 200 Increase™ 10/300”. The protein complex that eluted in the first peak was shown to consist of the pure StnABC variant (a), whereas the other peaks consisted of the same impurities quantified in Supplementary Fig. 1d, e. (c) Alignment of the C7 [4Fe4S]-cluster coordinating residues (amino acid 108-171 *S. ovata*) of potential Stn complexes found in other acetogens. (d) Superposition of FAD containing domain in StnC with NfnB. The R239 in StnC slightly switches the position which results in no hydrogen bonding with N5 nitrogen of FAD. The adjacent C7 [4Fe4S]-cluster to FAD in StnC is coordinated by K170, whereas in NfnB the same cluster had E126 as a ligand.

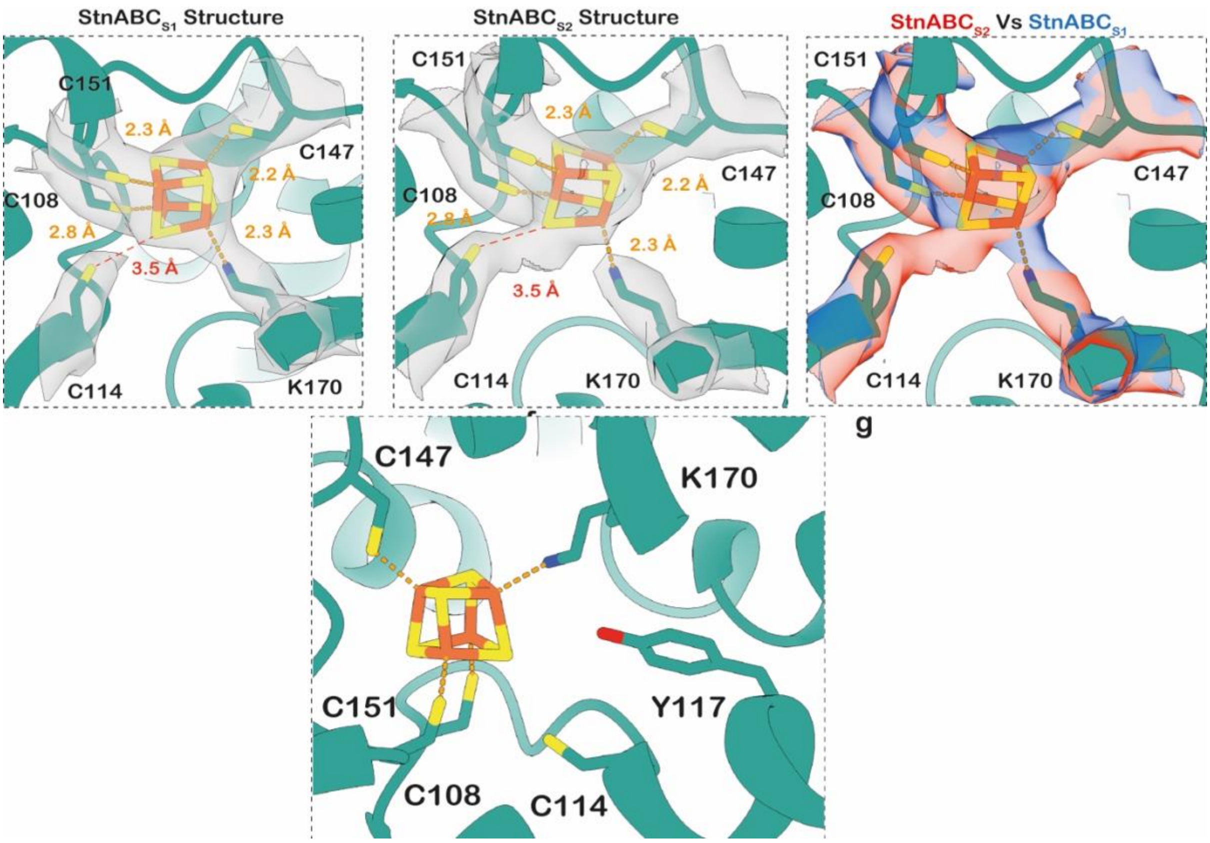

Supplementary Fig. 9 | Dynamics associated with the K170 and C114 of the C7 [4Fe4S]-cluster. Prominent density for lysine (K170) coordination in the C7 cluster was found in the StnABC$_{S1}$ state. Whereas, in the StnABC$_{S2}$ state with FMN-NAD^{+} bound, the cysteine (C114) coordination was observed, possibly indicating the formation of a hydrogen bond, where a proton is transferred from the neighbouring lysine. This condition of flipping the ligand coordination could alter the redox potential to promote directional electron transfer depending on the product formation.

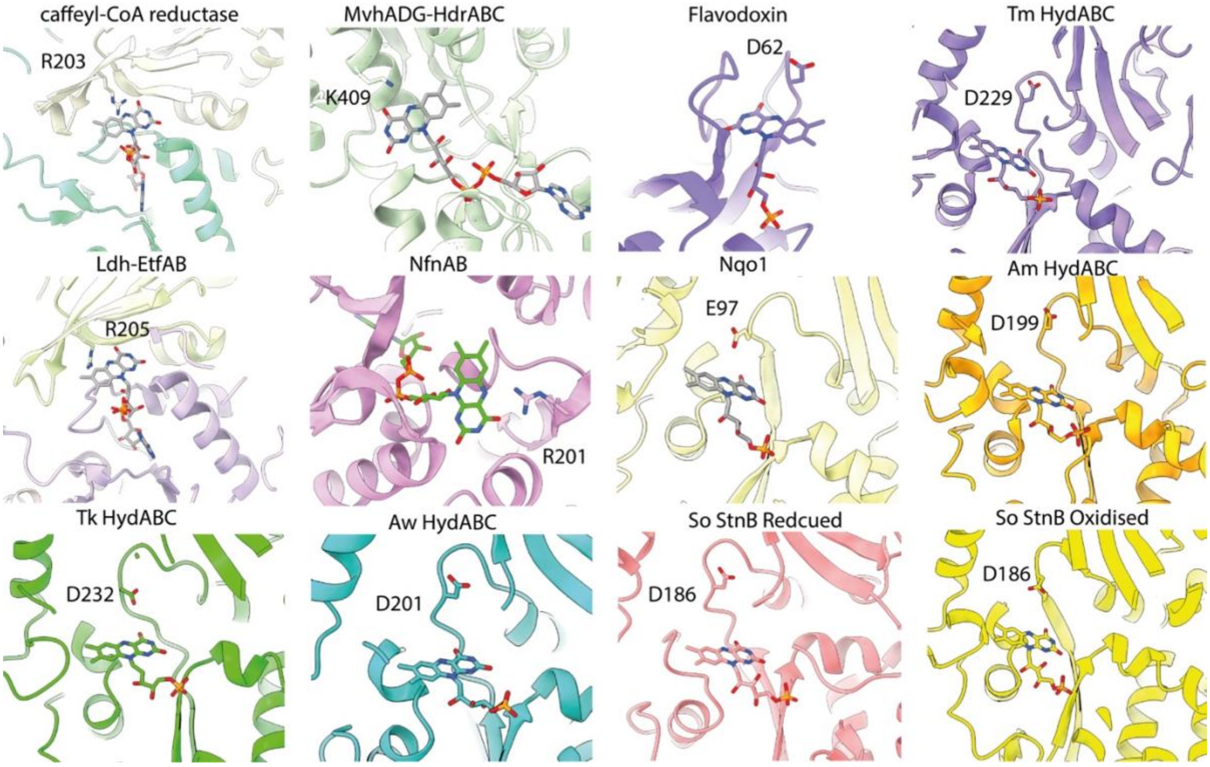

Supplementary Fig. 10 | Nearby residue modulating the function of electron bifurcating flavin. Classical bifurcating machines (Caffely-CoA reductase, NfnAB, HdrABC, and Ldh-EtfAB), which contain specifically an Arg or Lys residues that favour the formation of a short-living ASQ rather than an NSQ. In the case of recently discovered new class of electron bifurcating hydrogenase HydABC, such a scenario is absent and the flavin can only stabilize a neutral semiquinone with the help of Asp residue. This allows the flavin to act only as a mediator in the electron transfer process to nearby clusters. Flavin in Flavodoxin is coordinated by D62, which is also known for having a stabilized semiquinone, accepting or transporting electrons in a stepwise manner.

Supplementary Table 1 | Oxidoreductase activities of purified StnABC-variants. Activities are the mean of three independent biological replicates, measured in triplicates (n = 3). Activities are given in U/mg. One Unit is defined as the transfer of 2 µmol electrons/min.

Variant	NADPH:NAD$^+$:Fd_{ox} (340 nm) [U/mg]	NADPH:NAD$^+$:Fd_{ox} (430 nm) [U/mg]	NADH:Fd_{red}:NADP$^+$ (340 nm) [U/mg]	NADPH:MV_{ox} (604 nm) [U/mg]
**WT***	2.80 ± 0.57	1.59 ± 0.26	5.57 ± 1.37	164.08 ± 12.85
**StnABC-His**	1.08 ± 0.19	0.72 ± 0.29	1.35 ± 0.42	86.37 ± 16.62
**$K_{170}A$**	n.d.[1]	n.d.[1]	n.d.[1]	9.35 ± 2.85
**$K_{170}C$**	n.d.[1]	n.d.[1]	n.d.[1]	9.54 ± 0.86
**$K_{170}R$**	0.51 ± 0.10	0.25 ± 0.03	0.79 ± 0.09	47.01 ± 11.10
**$C_{114}A$**	0.56 ± 0.15	0.27 ± 0.13	0.74 ± 0.17	35.34 ± 8.85
**$R_{239}K$**	0.33 ± 0.10	0.21 ± 0.05	0.63 ± 0.21	65.42 ± 16.57
**$R_{239}A$**	0.03 ± 0.02	0.02 ± 0.01	n.d.[1]	5.38 ± 1.37
**ΔA1**	n.d.[1]	n.d.[1]	n.d.[1]	89.72 ± 19.51

[1]n.d., activity was not detectable.
*values represent a technical triplicate.

Supplementary Table 2 | Cryo-EM data collection, refinement and validation statistics.

	**StnABC** **$StnABC_{S1}$ state** (EMDB - 16879) (PDB - 8OH9)	**StnABC** **$StnABC_{S2}$ state** (EMDB - 16878) (PDB - 8OH5)
**Data collection and processing**		
Magnification	22,500 x	60,000 x
Voltage (kV)	300	200
Electron exposure (e–/Å^2)	55.0	50.0
Defocus range (μm)	0.8-1.8	1.2-1.8
Pixel size (Å)	1.09	0.84
Symmetry imposed	D2	D2
Initial particle images (no.)	326306	96166
Final particle images (no.)	116573	54596
Map resolution (Å)	3.2	3.0
FSC threshold	0.143	0.143
Map resolution range (Å)	2.7-4.0	2.5-4.0
**Refinement**		
Initial model used (PDB code)	*de novo*, AlphaFold	*de novo*, AlphaFold
Model resolution (Å)	3.4	3.2
FSC threshold	0.5	0.5
Model resolution range (Å)	3.1-3.6	3.2-3.6
Map sharpening *B* factor (Å^2)	-150.9	-88.4
Model composition		
Non-hydrogen atoms	58132	58624
Protein residues	7624	7624
Ligands	FMN: 0, NAP: 0, NDP: 0, FAD: 4, FES: 12, SF4: 36, Zn: 4	FMN: 4, NAI: 4, NDP: 4, FAD: 4, FES: 12, SF4: 36, Zn: 4
B factors (Å^2)		
Protein	50.04	12.80
Ligand	62.46	22.86
R.m.s. deviations		
Bond lengths (Å)	0.008	0.009
Bond angles (°)	0.967	0.884
Validation		
MolProbity score	1.68	1.54
Clashscore	5.17	5.01
Poor rotamers (%)	0.41	0.55
Ramachandran plot		
Favored (%)	96.45	96.54
Allowed (%)	3.27	3.10
Disallowed (%)	0.28	0.36

Supplementary Table 3 | List of cofactors (Prosthetic groups) and substrates found in the cryo-EM structure of StnABC complex its $StnABC_{S1}$ and $StnABC_{S2}$ states.

**Protein complex state**	**Cofactors (Prosthetic groups)**	**Substrates**
**$StnABC_{S1}$ state structure**	[4Fe4S], [2Fe2S], Zn^{2+} ion, FAD	NA
**$StnABC_{S2}$ state structure**	[4Fe4S], [2Fe2S], Zn^{2+} ion, FAD, FMN	NADPH, NAD^+, Fd

NA – not available.

The structure of the $StnABC_{S1}$ state was determined by mildly crosslinking the isolated or purified protein complex and subjecting it to cryo-EM. Here, no substrates (NADPH, NAD^+, and Fd) and cofactor (FMN) were added. Whereas, the structure of the $StnABC_{S2}$ state was obtained by mildly crosslinking the purified enzyme in the presence of exogenous substrates and cofactor (10 µM FMN and 500 µM NADPH, 500 µM NAD^+ and 30 µM Fd). The FAD was always added to the buffer while protein purification (see methods). The Fd could not be captured in our structure possibly due to rapid and transient binding and, therefore, is mentioned as a product.

Supplementary Table 4 | Oligonucleotides used in this study.

Name	Number	Sequence (5'->3')
**StnA_g_f**	1	TTTAAGAAGGAGATATACATATGTGTAATTCGTGCGAAAAAG
**StnB_g_r**	2	TATATTTATACTAATTTTGCTCATTATTCACCACTCCCTTCTAC
**pET21a_lin_f**	3	ATGTATATCTCCTTCTTAAAGTTAAAC
**pet21a_StnC_g_r**	4	ATGAGCAAAATTAGTATAAATATAAATGGC
**StnC_adhis_f**	5	CACCACCACTAGGGATCCGAATTCGAG
**StnC_adhis_r**	6	ATGATGATGCAAACTCTGCCTACCATTTAC
**StnC_ΔC1_f**	7	GCCGGCGTAGGCGCCAACGTTAATATAG
**StnC_ΔC1_r**	8	GTAGCCGGCAACCGAGGCCAT
**StnC_ΔC2_f**	9	TGGCCAGGCTGTCGATGTCTGTC
**StnC_ΔC2_r**	10	GCGGATATAGCAGCTGTCTCACTTAGCG
**StnC_K170C_f**	11	TGCTCAATTATGTAGCTTTGTTGC
**StnC_K170A_f**	12	TGCTCAATTAGCGAGCTTTGTTGC
**StnC_K170R_f**	13	TGCTCAATTAAAGAGCTTTGTTGC
**StnC_K170X_r**	14	ATAGAAATTGGCTCTTCCACC
**StnC_C114A_f**	15	CGCAAACCGATGCCCAGGGCTAT
**StnC_C114A_r**	16	CAGGACATGCCAGCATACATGGC
**StnC_R239A_f**	17	CGCAGTACGCGTTAGACAAAGC
**StnC_R239K_f**	18	CGCAGTACAAGTTAGACAAAGC
**StnC_R239X_r**	19	GAATACCGTAACGCAGCATACC
**StnC_ΔFdh_r**	20	ATGATGATGAACCGAGGCCATGCTGTCCATATTGG
**StnA_ΔA1_f**	21	CATGTACTTGGTTCTAAGGCGATAGGCAGTGC
**StnA_ΔA1_r**	22	AGCGGCTGTACCCTGAGCCTGCAGGATAAC

Supplementary Table 5 | Plasmids generated during this study.

**Plasmid**	**Description**	**Number of oligonucleotides used[1]**	**Introduced mutations**
**pET21a_StnABC-His**	WT Stn, C-terminal His-tag fused to StnC	1+2, 3+4, 5+6	None
**pET21a_StnΔA1**	Deletion of FeS cluster A1	21+22	C93A, C98A
**pET21a_StnK170A**	Exchange of C7 coordinating K170 to A170 in StnC	12+14	K170A
**pET21a_StnK170C**	Exchange of C7 coordinating K170 to C170 in StnC	11+14	K170C
**pET21a_StnK170R**	Exchange of C7 coordinating K170 to R170 in StnC	13+14	K170R
**pET21a_StnC114A**	Exchange of C114 to A in StnC	15+16	C114A
**pET21a_StnR239K**	Exchange of FAD-binding R239 to K239 in StnC	18+19	R239K
**pET21a_StnR239A**	Exchange of FAD-binding R239 to A239 in StnC	17+19	R239A
**pET21a_StnΔC1**	Deletion of FeS cluster C1	7+8	C692A, C695A, C699A
**pET21a_StnΔC2**	Deletion of FeS cluster C2	9+10	C656A, C659A, C662A
**pET21a_StnΔFdh**	Deletion of the Fdh domain	5+20	Truncated $StnC_{1-691}$, deletion of amino acid 692-1172

[1]for the sequence of oligonucleotides, see Table 4.

Supplementary Movie 1 | Overall representation of the segmented cryo-EM density map of the Stn tetramer with its corresponding atomic model and the functional StnABC protomer exhibiting the modular nature of the complex.

Colour codes are according to Fig. 1.
