# Supplementary material for: Molecular architecture and electron transfer pathway of the Stn family transhydrogenase
Source: Nat Commun. 2023 Sep 7;14:5484. doi: 10.1038/s41467-023-41212-x (PMC10482914; doi:10.1038/s41467-023-41212-x)
Supplement: Supplementary file 3 — Description of Additional Supplementary Files [file 41467_2023_41212_MOESM3_ESM.pdf]

File name: Supplementary Movie 1

Description: Overall representation of the segmented cryo-EM density map of the Stn tetramer with its corresponding atomic model and the functional StnABC protomer exhibiting the modular nature of the complex. Colour codes are according to Figure 1.
