# Supplementary material for: Molecular architecture and electron transfer pathway of the Stn family transhydrogenase
Source: Nat Commun. 2023 Sep 7;14:5484. doi: 10.1038/s41467-023-41212-x (PMC10482914; doi:10.1038/s41467-023-41212-x)
Supplement: Supplementary file 5 — Reporting Summary [file 41467_2023_41212_MOESM5_ESM.pdf]

nature portfolio

Corresponding author(s): Volker Müller, Jan Schuller

Last updated by author(s): 2023/08/22

Reporting Summary

Nature Portfolio wishes to improve the reproducibility of the work that we publish. This form provides structure for consistency and transparency in reporting. For further information on Nature Portfolio policies, see our Editorial Policies and the Editorial Policy Checklist.

Statistics

For all statistical analyses, confirm that the following items are present in the figure legend, table legend, main text, or Methods section.

n/a	Confirmed	
☐	☒	The exact sample size (*n*) for each experimental group/condition, given as a discrete number and unit of measurement
☐	☒	A statement on whether measurements were taken from distinct samples or whether the same sample was measured repeatedly
☒	☐	The statistical test(s) used AND whether they are one- or two-sided<br>*Only common tests should be described solely by name; describe more complex techniques in the Methods section.*
☒	☐	A description of all covariates tested
☒	☐	A description of any assumptions or corrections, such as tests of normality and adjustment for multiple comparisons
☐	☒	A full description of the statistical parameters including central tendency (e.g. means) or other basic estimates (e.g. regression coefficient) AND variation (e.g. standard deviation) or associated estimates of uncertainty (e.g. confidence intervals)
☒	☐	For null hypothesis testing, the test statistic (e.g. *F*, *t*, *r*) with confidence intervals, effect sizes, degrees of freedom and *P* value noted<br>*Give P values as exact values whenever suitable.*
☒	☐	For Bayesian analysis, information on the choice of priors and Markov chain Monte Carlo settings
☒	☐	For hierarchical and complex designs, identification of the appropriate level for tests and full reporting of outcomes
☒	☐	Estimates of effect sizes (e.g. Cohen's *d*, Pearson's *r*), indicating how they were calculated

Our web collection on statistics for biologists contains articles on many of the points above.

Software and code

Policy information about availability of computer code

Data collection	Size exclusion chromatography data was collected using UNICORN 5.31 software. WinASPECT 2.5.0.0 software was used for collecting photometric data.
Data analysis	Cryo electron microscopy data was analyzed with CryoSparc software. For analysis and image generation of protein structure, UCSF ChimeraX and PyMOL software was used. For model building and refinement, Coot and PHENIX software packages and tools were used. Prediction of structural models was performed using AlphaFold software. Quality assessment of the protein model was performed using PHENIX. GSFSC analysis was performed using CryoSparc. Size exclusion chromatography data was analyzed using UNICORN 5.31 software. WinASPECT 2.5.0.0 software was used for analyzing photometric data. The mass photometry was performed using OneMP mass photometer, and data was collected using AcquireMP.

For manuscripts utilizing custom algorithms or software that are central to the research but not yet described in published literature, software must be made available to editors and reviewers. We strongly encourage code deposition in a community repository (e.g. GitHub). See the Nature Portfolio guidelines for submitting code & software for further information.

Data

Policy information about availability of data

All manuscripts must include a data availability statement. This statement should provide the following information, where applicable:

- Accession codes, unique identifiers, or web links for publicly available datasets
- A description of any restrictions on data availability
- For clinical datasets or third party data, please ensure that the statement adheres to our policy

Cryo-EM maps are available in the Electron Microscopy Data Bank; StnABC state 2 (StnABCS2) (16878) and StnABC state 1 (StnABCS1) (16879). The atomic models of StnABC are available in the Protein Data Bank; StnABC state 2 (StnABCS2) (8OH5) and StnABC state 1 (StnABCS1) (8OH9). Structural and sequence data used for comparison with StnABC subunits are available in the Protein Data Bank (PDB - 6Q8W, NADH:quinone oxidoreductase from T. thermophilus; PDB - 5JCA, NADP(H) bound NADH-dependent Ferredoxin:NADP Oxidoreductase (NfnAB) from Pyrococcus furiosus; PDB - 7NZ1, NADH:ubiquinone oxidoreductase from Escherichia coli; PDB - 8A6T, electron bifurcating Fe-Fe hydrogenase HydABC complex from Thermoanaerobacter kivui; PDB ID - 1H0H, Tungsten containing Formate Dehydrogenase from Desulfovibrio Gigas; PDB ID - 3FX2, crystal structure of Flavodoxin. Strains and plasmids generated in this study are available from the corresponding authors upon request.

Human research participants

Policy information about studies involving human research participants and Sex and Gender in Research.

Reporting on sex and gender	Not applicable
Population characteristics	Not applicable
Recruitment	Not applicable
Ethics oversight	Not applicable

Note that full information on the approval of the study protocol must also be provided in the manuscript.

Field-specific reporting

Please select the one below that is the best fit for your research. If you are not sure, read the appropriate sections before making your selection.

☒ Life sciences ☐ Behavioural & social sciences ☐ Ecological, evolutionary & environmental sciences

For a reference copy of the document with all sections, see nature.com/documents/nr-reporting-summary-flat.pdf

Life sciences study design

All studies must disclose on these points even when the disclosure is negative.

Sample size	Sample size was not predetermined by statistical methods but sample sizes of n=3 or more were used for in vitro analysis to provide data sets that enable statistics for the relevant experiments. The in vitro experiments are tried-and-tested procedures that have been successfully performed in the author´s laboratories day-to-day for many years. The sample sizes were diligently chosen and accurately illustrate the differences throughout the various conditions. For cryo-EM, a data set containing 3000 micrographs were used in order to have a sufficient number of StnABC particles, which resulted in a 3D reconstruction with a resolution of 3.0 Angstrom. The achieved resolution was enough to model the protein structure at the residue level.
Data exclusions	For enzymatic assays of strictly anaerobic enzymes, oxygen contamination cannot always be excluded and leads to a significant reduction of enzymatic activity. If a measurement was obviously differing from the other replicates, this measurement was excluded from the data set. For cryo-EM, tetrameric StnABC complex were chosen for determination of the high resolution structure. Other particles were excluded from the analysis.
Replication	Enzymatic assays were performed in biological triplicates with three technical repetitions each. Sample separation via polyacrylamide gels or size exclusion chromatography was performed at least in two independent biological repetitions, respectively. For cryo-EM analysis of 3000 micrographs from each data set were analyzed. All assays could be successfully replicated.
Randomization	Resolution Determination was carried out with two independent half sets randomly selected by the CryoSparc processing software according to the Gold-Standard FSC procedure. For all other experiments, randomization was not applicable, since in microbiological assays all parameters are tightly controlled and therefore covariates are not relevant to our study.
Blinding	Blinding was not relevant to our study since no higher order species were used in this study. Moreover we have repeated experiments in biological triplicates with 3 technical replicates, and all assays were reproducible.

Reporting for specific materials, systems and methods

We require information from authors about some types of materials, experimental systems and methods used in many studies. Here, indicate whether each material, system or method listed is relevant to your study. If you are not sure if a list item applies to your research, read the appropriate section before selecting a response.

Materials & experimental systems

n/a	Involved in the study
☒	☐ Antibodies
☒	☐ Eukaryotic cell lines
☒	☐ Palaeontology and archaeology
☒	☐ Animals and other organisms
☒	☐ Clinical data
☒	☐ Dual use research of concern

Methods

n/a	Involved in the study
☒	☐ ChIP-seq
☒	☐ Flow cytometry
☒	☐ MRI-based neuroimaging
